# Nursing students’ emotional empathy, emotional intelligence and higher education-related stress: a cross-sectional study

**DOI:** 10.1186/s12912-023-01607-z

**Published:** 2023-11-20

**Authors:** Jiashuang Xu, Lin Zhang, Qiqi Ji, Pengjuan Ji, Yian Chen, Miaojing Song, Leilei Guo

**Affiliations:** 1 School of Nursing, Liaoning Province, Jinzhou Medical University, No.40, Section 3, Songpo Road, Linghe District, Jinzhou City, P. R. China; 2 Department of Internal Medicine Nursing, School of Nursing, Wannan Medical College, 22 Wenchang West Road, Higher Education ParkAn Hui Province, Wuhu City, P. R. China

**Keywords:** Nursing students, Emotional empathy, Emotional intelligence, Higher education-related stress

## Abstract

**Background:**

Nursing is one of the critically important disciplines in healthcare. Due to its complexity and specificity, nursing students often face additional higher education-related stress. However, there is less research on higher education-related stress among nursing students. Therefore, the purpose of this study was to investigate the effects of emotional empathy and emotional intelligence on the dimensions of higher education-related stress among nursing students.

**Methods:**

This study utilized a multi-stage sampling design and was completed within the March-June 2023 period. A total of 1126 nursing students were recruited to complete the questionnaire. The self-administered questionnaire included the basic characteristics of the subjects, an emotional empathy scale, an emotional intelligence scale, and a higher education-related stress scale. One-way ANOVA/t-tests, correlation coefficients, and hierarchical linear regression analyses were used to explore the factors affecting higher education-related stress and the relationship between emotional empathy and emotional intelligence with the dimensions of higher education-related stress.

**Results:**

The mean (SD) scores of challenges and dissatisfaction dimensions of higher education-related stress among nursing students were 30.052 (7.466) and 8.270 (2.205), respectively. Emotional empathy was significantly and positively related to the challenges and dissatisfaction dimensions of higher education-related stress. Emotional intelligence was negatively correlated with the challenges dimension of higher education-related stress and positively correlated with the dissatisfaction dimension. Stratified multiple regression analyses revealed that nursing students' emotional empathy and emotional intelligence were significant predictors of the dimensions of higher education-related stress.

**Conclusion:**

Overall, emotional empathy and emotional intelligence were significantly correlated with all dimensions of higher education-related stress. Consequently, in future interventions, the Chinese government and education sector can develop nursing students' ability to use emotional empathy and emotional intelligence rationally through emotional regulation strategies and emotional intelligence courses, to reduce the higher education-related stress they experience.

## Background

The nursing profession is one of the crucial disciplines in healthcare with tremendous social responsibility and influence [1]. “Nursing students”, as vital contributors to the healthcare industry, carry the sacred mission of caring for life and serving society [2]. However, the higher education stage is not only a critical stage for nursing students to realize their dreams but also a time of trial and stress [3, 4].

Higher education-related stress refers to the stresses related to academics, career development, and life that students face when they are in higher education [5]. The higher education level is usually accompanied by greater academic, professional, social, and psychological stress than other educational levels [6, 7]. This stress usually stems from increased course loads, higher academic requirements, uncertainty about future career prospects, social expectations, and personal achievement goals [8–10]. Higher education-related stress typically includes two main dimensions: challenging stress and dissatisfying stress. Challenging stress relates primarily to the difficulties students face in pursuing academic, practical, and other educational goals, whereas dissatisfying stress is more often seen in their evaluations of their academic achievement, interpersonal relationships with faculty, classmates, and patients, as well as their overall educational experience and clinical practice [11–13]. These higher education-related stressors not only have an impact on the academic performance of nursing students but may also have long-term negative effects on their mental health and emotional state [14, 15]. Therefore, an in-depth understanding of the factors that influence higher education-related stress is essential. Understanding these factors will help us to provide targeted support and guidance that will help nursing students to better cope with the various stressors and achieve success in their academic and future career development.

Emotional empathy is the ability of an individual to perceive another person's emotions, mental states, or experiences as his or her feelings and to communicate those feelings to others [16]. Emotion regulation theory suggests that emotional empathy allows nursing students to better understand and identify with the positive and negative emotions of others; when others are happy, they can resonate with this joy; and when faced with the pain of others, they will also feel the same [17, 18]. Almudena et al. showed that students with high emotional empathy are likely to experience similar emotions of pain and discomfort to some extent when faced with scenarios in which a patient undergoes surgery, postoperative complications, chemotherapy, and other ailments, which can lead to additional emotional challenges and psychological stress for the individual [19]. Mauno et al. showed that emotional empathy can be considered a valuable personal resource in the nursing profession. However, as the demands and workload increase, emotional empathy may change from an asset to a weakness, ultimately leading to negative effects such as dissatisfaction and increased stress [20]. Hence, in examining the relationship between emotional empathy and the dimensions of higher education-related stress in nursing students, we need to gain a deeper understanding of this complexity and find ways to balance the potential stress associated with emotional empathy.

Emotional intelligence is an individual's ability to perceive, understand, manage, and express emotions and involves sensitivity to one's own and others' emotional states, as well as the ability to respond appropriately and regulate them in different situations [21]. Emotional intelligence theory suggests that emotional intelligence plays a key role in the areas of emotion regulation, interpersonal relationships, future career development, and mental health, and is one of the key elements of individual growth and success [22]. Of particular note, emotional intelligence is tied to higher education-related stress. According to Yildirim et al., nursing students with higher emotional intelligence can quickly find solutions and coping strategies to mitigate the negative effects of stress when faced with challenges, thus maintaining good mental health and academic achievement in higher education settings [23]. Social learning theory suggests that those students with higher emotional intelligence can build mutually supportive social networks in the face of stress, thereby mitigating the negative effects of higher education-related stress. However, the relationship between emotional intelligence and higher education-related stress is not static [24]. Therefore, it is crucial to explore in depth the impact of emotional intelligence on the dimensions of higher education-related stress among nursing students. By gaining a deeper understanding and examining this impact, we can implement practical interventions to help students better cope with stress, thereby promoting their holistic development in higher education.

**Fig. 1:**
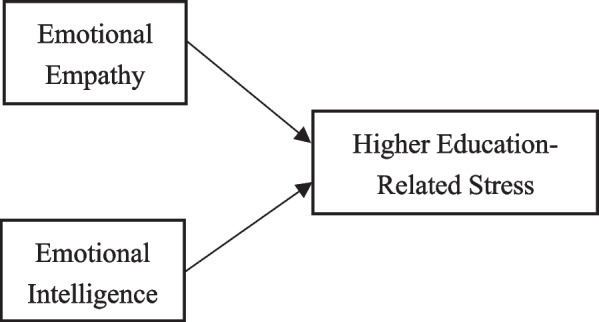
Hypothetical model

Based on published research, existing literature, and theory, we propose the following hypothesis: nursing students' emotional empathy and emotional intelligence are significantly correlated with all dimensions of higher education-related stress. (see Fig. 1). The purpose of this study is to provide a theoretical basis for how nursing students can effectively cope with higher education-related stress.

## Methods

### Design

This study employed a cross-sectional correlational design to investigate the relationship between emotional empathy, emotional intelligence, and the various dimensions of higher education-related stress in nursing students.

#### Participants and data collection

This study utilized a multi-stage sampling design to obtain participants. Inclusion Criteria: (1) enrolled as a full-time nursing student; (2) voluntary participation; and (3) ability to speak, read, and communicate effectively with the investigator. Exclusion Criteria: (1) Unwillingness to participate in the study. Based on the criteria proposed by Kendall, which require a minimum of 10 respondents per item [25], we calculated the sample size by multiplying the total number of items on the scale by 10 (N = [30 + 24 + 16] * 10 = 700). A total of 1186 questionnaires were administered face-to-face to participants by trained graduate nursing students, and 1176 were completed. After data cleaning, only 1126 forms (or a 94.94% response rate) were considered suitable for data analysis. The normality test showed that all data conformed to a normal distribution. The total number of participants met Kendall's sample size criteria.

This study was completed between March and June 2023. First, Jinzhou Medical University was randomly selected from 6 nursing schools in Liaoning Province. Second, 80% of the classes were sampled from each grade level (16 to 18 classes) of the university, including high school undergraduates and vocational college undergraduates. Thus, a total of 40 classes were sampled from the entire school. Thirdly, 25–30 students were selected from each class. A total of 1186 nursing students were sampled. Before data collection, the 10 graduate nursing students responsible for data collection received uniform training in the use of standardized language and instructions.

### Ethical considerations

This study was approved by the Ethical Review Committee of Jinzhou Medical University under the approval number JZMULL2023028. The study was initiated after informed consent was obtained from all participants. Throughout the study, we strictly adhered to the principles outlined in the Declaration of Helsinki to ensure the anonymity and confidentiality of participants' information and data.

### Instruments

The instrument used in this study was divided into two parts. Part 1 obtained demographic characteristics including gender, age, place of residence, grade level, willingness, and whether to engage in nursing after graduation. Gender was categorized as male and female. Age was categorized into three groups: 17–20, 21–25, and 26–30. Residence was categorized into three groups: urban, rural, and suburban. Grade level was categorized into three groups: freshman, sophomore, and junior. Willingness was categorized into three groups: voluntary, influenced by others, and other. Whether to engage in nursing after graduation was categorized into three groups: yes, no, and undecided.

Part 2 contains 3 standardized scales: the 30-item Multidimensional Emotional Empathy Scale, the 24-item Emotional Intelligence Scale, and the 16-item Higher Education Stress Inventory.

#### Emotional empathy

The 30-item Multidimensional Emotional Empathy Scale developed by Caruso and Mayer was used [26]. The scale consists of 30 questions that cover multiple aspects of an individual's emotional empathy skills. A Likert score was used on the scale where participants were required to score between 1 (strongly disagree) and 5 (strongly agree). The scale is organized into six dimensions: Suffering: Items 3, 5, 6, 8, 12, 18, 24, 28. Positive Sharing: Items 14, 22, 23, 29, 30. Responsive Crying: Items 1, 20, 25. Emotional Attention: Items 4, 9, 13, 27. Feelings for Others: Items 2, 10, 15, 16. Emotional Contagion: Items 11 and 17. The total score on the scale ranges from 30 (lowest) to 150 (highest). In this study, the Cronbach's alpha coefficient for this scale was 0.780.

#### Emotional intelligence scale

The 24-item Emotional Intelligence Scale, developed by American psychologists John D. Mayer and Peter Salovey in 1990, is designed to assess an individual's emotional intelligence [27]. This scale comprises 24 items distributed across three dimensions. Specifically, items 1 to 8 pertain to the Emotional Perception dimension, items 9 to 16 relate to the Emotional Understanding dimension, and items 17 to 24 belong to the Emotional Regulation dimension. The scoring ranges for each dimension are as follows: For the Emotional Perception dimension: A low range (men: < 21; women: < 24) suggests a need for improvement in emotional perception. Moderate range (men: 22 to 32; women: 25 to 35) indicates sufficient emotional perception abilities. The high range (men: > 33; women: > 36) implies highly developed emotional perception skills. For the Emotional Understanding dimension: A low range (men: < 25, women: < 23) indicates a need for improvement in emotional understanding. Moderate range (men: 26–35; women: 24–34) signifies adequate emotional understanding abilities. The high range (men: > 36; women: > 35) suggests outstanding emotional understanding skills. For the Emotional Regulation dimension: A low score (men and women: < 23) suggests a need for improvement in emotional regulation. A moderate score (men: 24–35; women: 24–34) indicates sufficient emotional regulation abilities. A high score (men: > 36; women: > 35) signifies strong emotional regulation capabilities. Participants employ a Likert five-point scale to rate their agreement with each item, ranging from 1 (strongly disagree) to 5 (strongly agree). The total score is obtained by summing the scores from each dimension, with each dimension's score ranging from 8 to 40. In this study, the Cronbach's α coefficient for this scale was found to be 0.865.

#### Higher education-related stress scale

The 16-item Higher Education Stress Inventory was developed by Dahlin et al. to measure higher education-related stress among medical students [28]. The scale is neutral to the educational environment and can be used for comparisons between different student populations. The scale consists of 16 items categorized into 2 dimensions: challenge and dissatisfaction. 1–13 is the challenge dimension and 14–16 is the dissatisfaction dimension. The items are scored on a 4-point Likert scale where 1 = completely disagree, 2 = somewhat disagree, 3 = somewhat agree, and 4 = completely agree. The Cronbach's alpha for this scale in this study was 0.794. Through the use of the HESI scale, researchers can gain a deeper understanding of the various stressors that medical students may face in a higher education setting, thus providing useful information for improving the educational experience and providing support programs.

#### Statistical analysis

Differences in categorical variables for higher education-related stress were tested by independent samples t-tests and one-way ANOVAs. Pearson correlation analysis is used to test the correlation between continuous variables. Separate hierarchical multiple regression analyses were conducted to test the incremental variance of any given set of independent variables, using each of the dimensions of higher education-related stress as the dependent variable. In this study, the characteristics of nursing students were added to the regression model in the first step, emotional empathy in the second step, and emotional intelligence in the third step. The relative importance of the variables retained in the final multiple regression model helps to explain the variance of the dimensions in higher education-related stress, which is denoted as standardized beta. Adjusted *R*^*2*^ values were used to assess model fit. *p* < 0.05 (bilateral) were considered statistically significant. All data were analyzed using IBM SPSS version 25.0 (IBM Corp, Armonk, NY).

## Results

### Participant characteristics

Valid responses were obtained from 1126 nursing students. Table 1 provides the basic characteristics of the participants. The mean ± SD age of the participants was 21.82 ± 1.47 years, ranging from 17 to 30 years. The majority of participants (*N* = 981, 87.1%) were female. As for residential status, the majority of participants 53.0% lived in urban areas, 38.7% in rural areas, and 8.3% in suburban areas. As for the willingness to choose the nursing program, 56.8% of all participants chose the nursing program voluntarily, 24.2% were influenced by others to choose the nursing program, and 19.0% chose the nursing program for other reasons. When it comes to whether or not to pursue a nursing profession after graduation, 47.4% of the participants answered yes, 12.0% answered no, and 40.6% answered unsure. More details are shown in Table 1.

**Table 1:** Characteristics of nursing students, means and standard deviations of challenges and dissatisfaction (*N* = 1126)

Variables	N(%)	Challenge (Mean ± SD)	dissatisfaction (Mean ± SD)
**Total**	1126	30.05 ± 7.47	8.27 ± 2.20
**Gender**			
Male	145(12.9)	30.32 ± 8.11	7.96 ± 2.63
Female	981(87.1)	30.01 ± 7.37	8.31 ± 2.13
**Age**			
17–20	194(17.2)	30.76 ± 6.99	8.07 ± 2.20
21–25	927(82.3)	29.88 ± 7.55	8.30 ± 2.20
≥ 26	5(0.4)	33.60 ± 7.23	8.60 ± 2.40
**Place of residence**			
Urban	597(53.0)	29.15 ± 7.88 ^***^	8.27 ± 2.36
Rural	436(38.7)	31.21 ± 6.79	8.24 ± 1.99
Suburban	93(8.3)	30.40 ± 7.06	8.36 ± 2.09
**Grade**			
Freshman	511(45.4)	30.26 ± 7.74	8.27 ± 2.24
Sophomore	460(40.9)	29.90 ± 6.88	8.35 ± 2.20
Junior	155(13.8)	29.81 ± 8.22	7.97 ± 2.08
**Willingness**			
Voluntary Choice	640(56.8)	29.16 ± 7.52 ^***^	8.46 ± 2.21 ^***^
Influenced by others	272(24.2)	31.36 ± 7.40	8.12 ± 2.18
Others	214(19.0)	31.03 ± 7.04	7.87 ± 2.14
**Whether to engage in nursing after graduation**			
Yes	534(47.4)	29.30 ± 7.61 ^**^	8.63 ± 2.16 ^***^
No	135(12.0)	31.04 ± 7.53	7.77 ± 2.38
Uncertain	457(40.6)	30.63 ± 7.19	7.98 ± 2.12

^*^p < 0.05, ^**^p < 0.01, ^***^p < 0.001

### Description of HESI for nursing students

In this study, the mean ± SD scores of the challenges and dissatisfaction dimensions of higher education-related stress among nursing students were 30.05 ± 7.47and 8.26 ± 2.20, respectively, and the dissatisfaction dimension of higher education-related stress scores was significantly lower than the challenges dimension scores. When the dimension of challenges in higher education-related stress was the dependent variable, the differences in place of residence, willingness, and whether or not to pursue a nursing career after graduation were statistically significant among all variables. In contrast, when the dissatisfaction dimension of higher education-related stress was used as the dependent variable, of all the variables, only the differences in willingness and whether or not to pursue a nursing career after graduation were statistically significant (Table 1).

### Correlation of HESI variables with predictors for nursing students

In Pearson's correlation analysis, emotional empathy was significantly and positively correlated with the challenges dimension and dissatisfaction dimension in higher education-related stress. Whereas emotional intelligence was significantly negatively correlated with the challenges dimension and significantly positively correlated with the dissatisfaction dimension in higher education-related stress. See Table 2.

**Table 2:** Means, standard deviations and correlations among the study variables

Variables	Mean ± SD	1	2	3	4
Emotional Empathy	87.667 ± 15.276	1			
emotional intelligence	91.696 ± 15.261	0.319^**^	1		
challenges	30.052 ± 7.466	0.086^**^	-0.100^**^	1	
dissatisfaction	8.270 ± 2.205	0.195^**^	0.254^**^	0.242^**^	1

^**^P < 0.01

### Hierarchical multiple regression analysis

The results of the hierarchical multiple regression models for the challenge and dissatisfaction dimensions of higher education-related stress are presented in Tables 3 and 4. Each step of the independent variable contributes significantly to the variance of the challenge and dissatisfaction dimensions of higher education-related stress. When using the challenges dimension of higher education-related stress as the dependent variable, in the first step, demographic characteristics, including gender, age, place of residence, grade level, willingness, and whether or not to pursue a nursing career after graduation, as a whole, explained 2.5% of the variation in challenges. In a second step, after controlling for demographic characteristics, this study found that emotional empathy was significantly and positively related to the challenges dimension of higher education-related stress. Emotional empathy had a significant effect on the challenges dimension of higher education-related stress among nursing students (*F* = 6.229, adjusted* R*^*2*^ = 0.038, *R*^*2*^ change = 0.008). In the third step, emotional intelligence was added to the model and it was significantly negatively correlated with the challenges dimension of higher education-related stress. This independent variable explained 1.3% of the variation in the challenges dimension of higher education-related stress. In addition, all variables in the model explain 4.4% of the variance in the challenges dimension of higher education-related stress; When using the dissatisfaction dimension of higher education-related stress as the dependent variable, in the first step, demographic characteristics explained 2.5% of the variance in the dissatisfaction dimension of higher education-related stress. In a second step, after controlling for demographic characteristics, this study found that emotional empathy was significantly and positively associated with the dissatisfaction dimension of higher education-related stress. Emotional empathy had a significant effect on the dissatisfaction dimension of higher education-related stress among nursing students (*F* = 11.673, adjusted *R*^*2*^ = 0.062, *R*^*2*^ change = 0.038). In the third step, emotional intelligence was added to the model and it was significantly and positively correlated with the dissatisfaction dimension in higher education-related stress and this independent variable explained 3.5% of the variance in the dissatisfaction dimension in higher education-related stress. All variables in the model explain 9.7% of the variance in the dissatisfaction dimension of higher education-related stress.

**Table 3:** Hierarchical multiple regression prediction challenge scores

Variables	Step 1		Step 2		Step 3	
	*β*	*P*	*β*	*P*	*β*	*P*
Control variables						
Sex	-0.010	0.733	-0.013	0.655	-0.008	0.789
Age	-0.033	0.262	-0.037	0.210	-0.036	0.225
Place of residence	0.109^***^	< 0.001	0.110^***^	< 0.001	0.100^***^	0.001
Grade	-0.016	0.596	-0.015	0.603	-0.020	0.488
Willingness	0.096^**^	0.002	0.096^**^	0.002	0.088^**^	0.004
Whether to engage in nursing after graduation	0.061*	0.049	0.059	0.054	0.055	0.074
Emotional Empathy			0.087^**^	0.003	0.126^***^	< 0.001
Emotional intelligence					-0.123^***^	< 0.001
*F*	5.752^***^		6.229^***^		7.475^***^	
*R*^*2*^	0.030		0.038		0.051	
Adjusted *R*^*2*^	0.025		0.032		0.044	
*R*^*2*^-change	0.030		0.008		0.013	

^*^ *p* < 0.05, ^**^ *p* < 0.01, ^***^p < 0.001

**Table 4:** Hierarchical multiple regression analysis results of dissatisfaction

**Variables**	**Step 1**		**Step 2**		**Step 3**	
	***β***	***P***	***β***	***P***	***β***	***P***
Control variables						
Sex	0.056	0.060	0.049	0.093	0.040	0.160
Age	0.008	0.799	-0.001	0.970	-0.003	0.905
Place of residence	-0.002	.946	-0.001	0.980	0.015	0.603
Grade	-0.046	0.122	-0.045	0.121	-0.037	0.196
Willingness	-0.067^*^	0.029	-0.068^*^	0.026	-0.055	0.066
Whether to engage in nursing after graduation	-0.129^***^	< 0.001	-0.132^***^	< 0.001	-0.125^***^	< 0.001
Emotional Empathy			0.196^***^	< 0.001	0.133^***^	< 0.001
Emotional intelligence					0.200^***^	< 0.001
*F*	5.714^***^		11.673^***^		16.126^***^	
*R*^*2*^	.030		0.068		0.104	
Adjusted *R*^*2*^	0.025		0.062		0.097	
*R*^*2*^-change	0.030		0.038		0.035	

^*^p < 0.05, ^**^ *p* < 0.01, ^***^p < 0.001

## Discussion

To the best of our knowledge, this study is the first to explore and identify the relationships between demographic characteristics, emotional empathy, and emotional intelligence with various dimensions of higher education-related stress among nursing students. This study is valuable for understanding how nursing students cope with higher education-related stress and maintain physical and mental health.

The results of our multiple linear regression analyses showed that nursing students' emotional empathy and emotional intelligence were significant predictors of the dimensions of higher education-related stress, implying that they were effective in predicting nursing students' performance in coping with higher education-related stress. As reported by Philip's newspaper, empathy is a significant predictor of stress along with depression and anxiety [29]. However, it is important to emphasize that although multiple linear regression analysis helps to determine that there is a relationship between the independent and dependent variables, it does not explain why this relationship exists.

In support of the results of the multiple linear regression analysis, recent research has shown that emotional empathy is a significant predictor of stress. Zhang et al. found that among nursing students, those with higher emotional empathy or those who described strong feelings of uneasiness and tension in interpersonal settings typically exhibited higher levels of stress [30]. Ferri et al.'s study confirmed that students with high emotional empathy are more likely to be touched by the pain and misery of their patients, which in turn induces emotions such as sadness and anxiety in students, increasing the emotional burden and challenging stress they face [31, 32]. Bidyadhar et al. found that students with high emotional empathy usually show high levels of emotional support and care when interacting with patients or individuals in need. However, excessive support and caring may trigger emotional exhaustion and fatigue and reduce their sensitivity to the emotional needs of others, which negatively affects self-satisfaction and ultimately leads to higher levels of stress [20, 33]. These findings support the results of this study and emphasize the value of the proper and rational use of emotional empathy in coping with higher education-related stress.

In addition, emotional intelligence was strongly associated with dimensions of higher education-related stress, which is consistent with previous findings. Alinejad et al. found that students with high emotional intelligence were more capable of handling challenging emotional encounters with patients and therefore felt less stress [34]. Rodríguez et al. showed that nursing students with high emotional intelligence may be more capable of maintaining emotional stability in complex and stressful clinical environments, which in turn enables them to use positive emotion regulation strategies, such as seeking support and developing solutions, when faced with challenges such as emergencies and contingencies, thereby reducing the difficulty of the challenge and decreasing the level of stress associated with the challenge [35]. Por et al. found that people with high emotional intelligence appear to be better at self-regulating their emotions and expressing them effectively while being able to accurately interpret the deeper emotional meanings of those with whom they interact. As a result, they are usually better able to cope with challenges and are less susceptible to stress, as well as having a lower risk of mental health problems [36]. In contrast, Mohammad et al. showed that nursing students with high emotional intelligence usually tend to be self-critical when coping with less-than-ideal or failing-to-meet-expectations situations, blaming their lack of competence, which in turn triggers dissatisfaction and leads to higher levels of stress [37]. A study by M Zakirulla et al. found that students with higher emotional intelligence usually have clear goal orientations for themselves, and they tend to invest more in academics and practices to reach these goals. However, too much investment can sometimes lead to additional emotional distress. For example, they may work continuously to achieve excellent grades or performance, but when the actual results do not match their expectations, it may trigger stronger feelings of dissatisfaction, which can lead to higher stress levels [38]. Collectively, the role of emotional intelligence in students' exposure to higher education-related stressors is complex and varied, which may be related to their ability to regulate emotions, perceive emotions, and understand emotions [39]. This finding highlights the importance of emotional intelligence in education. It provides a theoretical basis for facilitating students' better adaptation and success in facing higher education-related stress.

In addition to this, many studies have emphasized the critical role of nursing education, clinical faculty, nursing curricula, and nursing schools in reducing higher education-related stress in nursing students. These studies recommended the inclusion of courses on emotion regulation, emotional intelligence, and stress management in the training of nursing students, arguing that these elements are critical in reducing higher education-related stress [40–42]. Therefore, it is the responsibility of relevant government departments and nurse educators to develop nursing students' competence in understanding and applying emotional empathy and emotional intelligence so that they can effectively cope with and overcome higher education-related stressors and various potential barriers and adversities.

### Limitations

Our study has several limitations. First, due to the cross-sectional design and self-report measures, it was not possible to conclude the causal relationship between emotional empathy, emotional intelligence, and higher education-related stress. In future research, longitudinal studies are needed to further explore the relationship between these variables. Second, the sample of our study was nursing students from a specific city, which may not fully represent the actual situation of the entire nursing student population. Future studies may consider expanding the sample to cover more regions and different types of nursing educational institutions to increase the applicability and breadth of the findings.

### Recommendations

Based on the results of this study, we offer some enlightening suggestions. First, the Chinese government and education authorities should emphasize the development of nursing students' ability to use emotional empathy rationally. For example, activities such as role-playing, practical application of emotion regulation strategies, and case studies related to clinical practice should be encouraged. This will help students who may need additional help in emotional processing to better cope with challenges and emotional distress, thereby reducing sources of stress [43] Secondly, more emphasis should be placed on developing students' emotional intelligence by offering courses on emotional intelligence such as emotion recognition and stress coping skills [44]. These courses will help them to better manage their emotions and properly deal with the challenges and dissatisfaction associated with their studies and internships, thus reducing the level of stress. In conclusion, this study provides valuable insights into the relationships between emotional empathy, emotional intelligence, and various dimensions of higher education-related stress among nursing students. These findings not only offer guidance for improving nursing education but also offer substantial recommendations for students to better cope with stress-related issues.

## Conclusions

Overall, emotional empathy and emotional intelligence were significantly associated with all dimensions of higher education-related stress. Therefore, future interventions should specifically focus on developing nursing students' ability to use emotional empathy and emotional intelligence rationally. Providing relevant education and training will help students reduce feelings of stress associated with challenges and dissatisfaction and maintain the physical and mental health of individuals.

## Data Availability

The datasets used and analyzed during the current study are available from the corresponding author upon reasonable request.

## Abbreviations

SD: Standard Deviation
HESI: Higher education-related stress Scale
β: Standardized
